# Editorial: AI and multi-omics for rare diseases: challenges, advances and perspectives, Volume III

**DOI:** 10.3389/fmolb.2024.1392943

**Published:** 2024-03-12

**Authors:** Frank Emmert-Streib, Silvia Bottini, Leonardo Franco

**Affiliations:** ^1^ Predictive Society and Data Analytics Lab, Faculty of Information Technology and Communication Sciences, Tampere University, Tampere, Finland; ^2^ Center of Modeling, Simulation and Interactions, Université Côte d’Azur, Nice, France; ^3^ Computer Science Department, Malaga University, Malaga, Spain

**Keywords:** rare disease, artificial intelligence, omics, data science, high-throughput

In general, a rare disease (RD) is defined by its occurrence in a small fraction of the population. In Europe, a condition is classified as rare when it impacts fewer than 1 in 2000 citizens. Globally, there are over 7,000 recognized RDs. Despite being individually uncommon, RDs collectively affect an estimated 350 million individuals worldwide. Predominantly genetic in nature, most RDs persist throughout an individual’s lifetime, even if symptoms are not immediately apparent. RDs manifest a wide range of symptoms, which can vary significantly among patients. Furthermore, these symptoms may mimic those of more common diseases, often resulting in misdiagnosis.

To address ongoing challenges surrounding rare diseases, we organized a Research Topic entitled ”AI and Multi-Omics for Rare Diseases: Challenges, Advances, and Perspectives,” marking its third installment (Vol 1 and Vol 2). The Research Topic yielded a total of four articles. The first article authored by Bohn et al. delineates a meticulously compiled census of variants within the 3′ and 5′ UTR, emphasizing the mechanisms through which they induce pathogenic effects. Their analysis encompasses 295 3′ and 188 5′ UTR variants sourced from ClinVar, of which 26 3′ and 68 5′ UTR variants were categorized as either pathogenic (P) or likely pathogenic (LP). Notably, deep learning models exhibited statistically significant distinctions when contrasting model-aligned P/LP variants against both putatively benign variants and model-mismatched P/LP variants. Additionally, a significant disparity in PhyloP conservation scores between P/LP variants and putatively benign variants emerged in both the 3′ and 5′ UTR regions. In summary, the study identifies a robust set of P/LP 3′ and 5’ UTR variants and elucidates diverse underlying mechanisms supported by comprehensive curation of pathogenicity evidence and molecular mechanisms.

The work by Martin-Hernandez et al. analyzed a comprehensive multi-omics dataset including genes, microRNAs, and methylation sites. Leveraging sophisticated systems biology tools, the study found additional genes that govern the elements encapsulated in the multi-omics signature. Moreover, through RNA-seq, miRNA-seq, and DNA methylation profiling, the study demonstrated a remarkable ability to discriminate between stage I-II and stage III-IV patients, outperforming previously identified prognostic biomarkers. Validation with an independent dataset further confirmed the association of signature genes with Overall Survival (OS) data, revealing that patients exhibiting distinct expression patterns in 8 genes and 4 microRNAs experienced a statistically significant decrease in OS. Finally, the authors presented an autonomous prognostic signature tailored for ACC that is potentially transformative in the clinical setting. This amalgam of 9 gene/microRNA features effectively predicted high-risk ACC cancer patients, with promising implications for clinical application.

The article by Maitra et al. introduces a novel unsupervised method for integrating single-cell multi-omics data, termed UMINT (Unsupervised Multi-omics INTegration). UMINT, based on neural networks, enables the seamless integration of a variable number of single-cell omics layers characterized by high dimensions. The proposed model demonstrates the ability to learn a latent low-dimensional embedding, thereby extracting pertinent features from the data to facilitate subsequent downstream analyses. The efficacy of UMINT has been demonstrated through its application to the integration of diverse datasets, including healthy and diseased CITE-seq profiles (comprising paired RNA and surface proteins), notably encompassing a rare condition, Mucosa-Associated Lymphoid Tissue (MALT) tumors. Rigorous benchmarking against the prevailing state-of-the-art methodologies for single-cell multi-omics integration underscores UMINT’s superiority. Moreover, the versatility of UMINT extends to the integration of paired single-cell gene expression and ATAC-seq (Assay for Transposase-Accessible Chromatin) assays, further enhancing its utility in multiple domains of biological investigation.

The fourth paper by Choon et al. provides a review of the state of the art in artificial intelligence methods and databases for Next-Generation Sequencing (NGS)-based diagnosis of rare diseases. The paper also compares several rare disease databases. A particular challenge of clinical NGS for diagnosis emphasized in this paper is the interpretation of results. The problem is that this cannot be achieved in a mechanistic manner but requires statistical thinking.

In summary, the three volumes of our Research Topic on AI and Multi-Omics for Rare Diseases have published a total of 15 articles. This indicates a sustained interest and demand for further advancements in this field, driven in part by the continuous influx of new data and advances in AI methodologies capable of pushing boundaries.

